# Importance of Multiple Reinforcing Comments and Areas for Change in Optimizing Dietary and Exercise Self-Monitoring Feedback in Behavioral Weight Loss Programs: Factorial Design

**DOI:** 10.2196/18104

**Published:** 2020-11-23

**Authors:** Rebecca Krukowski, Hyeonju Kim, Melissa Stansbury, Qian Li, Saunak Sen, Gregory Farage, Delia West

**Affiliations:** 1 Department of Preventive Medicine College of Medicine University of Tennessee Health Science Center Memphis, TN United States; 2 Arnold School of Public Health University of South Carolina Columbia, SC United States; 3 Department of Psychology University of Memphis, Memphis Memphis, TN United States

**Keywords:** overweight, obesity, weight loss, feedback, diet records, compliance, counselor

## Abstract

**Background:**

Individualized dietary and physical activity self-monitoring feedback is a core element of behavioral weight loss interventions and is associated with clinically significant weight loss. To our knowledge, no studies have evaluated individuals’ perspectives on the composition of feedback messages or the effect of feedback composition on the motivation to self-monitor.

**Objective:**

This study aims to assess the perceptions of feedback emails as a function of the number of comments that reinforce healthy behavior and the number of areas for change (ie, behavioral changes that the individual might make to have an impact on weight) identified.

**Methods:**

Emailed feedback followed a factorial design with 2 factors (ie, reinforcing comments and areas for change), each with 3 levels (ie, 1, 4, or 8 comments). A total of 250 adults with overweight or obesity who were interested in weight loss were recruited from the Qualtrics research panel. Participants read 9 emails presented in a random order. For each email, respondents answered 8 questions about the likelihood to self-monitor in the future, motivation for behavioral change, and perceptions of the counselor and the email. A mixed effects ordinal logistic model was used to compute conditional odds ratios and predictive margins (ie, average predicted probability) on a 5-point Likert response scale to investigate the optimal combination level of the 2 factors.

**Results:**

Emails with more reinforcing comments or areas for change were better received, with small incremental benefits for 8 reinforcing comments or areas for change versus 4 reinforcing comments or areas for change. Interactions indicated that the best combination for 3 of 8 outcomes assessed (ie, motivation to make behavioral changes, counselor’s concern for their welfare, and the perception that the counselor likes them) was the email with 8 reinforcing comments and 4 areas for change. Emails with 4 reinforcing comments and 4 areas for change resulted in the highest average probability of individuals who reported being very likely to self-monitor in the future.

**Conclusions:**

The study findings suggest how feedback might be optimized for efficacy. Future studies should explore whether the composition of feedback email affects actual self-monitoring performance.

## Introduction

### Background

Individualized dietary and physical activity self-monitoring feedback on the frequency of monitoring, reinforcing comments about positive behaviors, and specific recommendations for behavioral change based on self-monitoring data is a core element of behavioral weight loss interventions [1-3]. Furthermore, Tate et al [4] found that human-generated emails produce significantly greater weight loss over 6 months than computer-automated feedback, which may reflect the benefit of tailored messages in general [5]. Despite the clear benefits of self-monitoring feedback in behavioral weight control programs, surprisingly little empirical information exists to guide the crafting of feedback messages. Some have suggested a formula for feedback, which *sandwiches* comments that reinforce positive health behaviors with suggested areas of change [6]. Others have argued that providing a *menu* of areas for change, which encompasses multiple dietary and physical activity behaviors to give individuals the option to choose what they would like to adopt, if any, is preferable to a prescriptive approach that targets one area to change [7].

To our knowledge, no studies have evaluated how individuals who attempt weight control perceive the effect of feedback messages on their self-monitoring or whether feedback composition affects the likelihood to self-monitor in the future or strengthens the relationship between the participant and the interventionist. As counselor time is one of the most expensive aspects of a behavioral weight management intervention [8,9], it is critical to examine whether longer, personalized feedback (requiring greater time to craft) would prompt continued self-monitoring and begin to identify the composition of feedback that would be expected to promote subsequent self-monitoring. Optimizing self-monitoring feedback may strengthen the working relationship between counselors and participants and, ultimately, increase weight loss.

From a theoretical perspective, feedback that reinforces positive health behaviors would be expected to build self-efficacy for behavioral change and provide positive outcome expectations for self-monitoring [10,11]. Given the strong positive association between greater self-monitoring and greater weight loss [12], outcome expectations that support self-monitoring and enhance self-efficacy for weight loss–promoting behaviors should promote better weight loss. Identifying multiple suggested areas for behavioral change supports autonomy because it gives the individual an option to select from these areas [13], and increased autonomy is also associated with better weight loss outcomes [14,15].

### Objectives

Therefore, the purpose of this study is to assess how individuals perceive self-monitoring feedback emails (ie, likelihood to self-monitor in the future, motivation to make behavioral changes, and perceptions of the counselor and emails) depending on the number of comments on positive health behaviors and the number of identified areas for change. This study also examines whether emails would be better received if they had a greater number of reinforcing comments than areas for change. We predicted that emails with the highest number of reinforcing comments (ie, 8) would be perceived as optimal for encouraging treatment engagement, but a moderate number of areas for change (ie, 4 areas for change) would be preferred.

## Methods

### Design

A total of 250 participants were recruited from the Qualtrics research panel, a web-based survey platform available to researchers to facilitate participant recruitment and web-based data collection, from April to May 2019 [16]. Qualtrics partners with more than 20 web-based panel providers; these panel partners randomly select respondents from their cadre of individuals and send them invitations to participate in surveys. Individuals completed a screening questionnaire to identify those who were eligible (ie, aged ≤18 years, had a BMI ≥25 kg/m^2^, and a desire to lose weight). To ensure a demographically balanced survey panel, the number of participants within the gender and race strata (ie, individuals who identified as White and those who identified as another racial group) was capped. Qualtrics seeks to identify fraudulent respondents when taking surveys by methods such as monitoring respondents’ survey speed and internet protocol addresses. Respondents were given point-based incentives by the panel provider that could be redeemed in various ways (eg, airline miles, credit for web-based games, and gift cards) for completing the survey. This study was approved by the institutional review board at the University of Tennessee Health Science Center, and participants provided informed consent.

Respondents were asked to imagine that they were in a weight loss program in which they recorded their diet and exercise daily, and they were told that they received feedback about their self-recorded diet and exercise from a counselor weekly via the following emails. Next, respondents read 9 emails in a computer-randomized order. The feedback presented in the emails followed a full factorial design with 2 factors (ie, reinforcing comments and areas for change), each with 3 levels (ie, 1 comment, 4 comments, and 8 comments).

After reading each email, respondents answered 8 questions about their likelihood to self-monitor in the future, motivation to make behavioral changes, and their perceptions of the counselor and the emails. Respondents spent an average of 27 (SE 3) min to complete the survey.

### Emails

Archived email feedback on diet and exercise self-monitoring that had been previously written for participants in the iREACH3 behavioral weight control study [17] was used in this study. A pool of emails was classified into points that noted positive behaviors and those that suggested an area of change to consider. A total of 9 emails were crafted to meet the requirements of the study (eg, one email that had 1 reinforcing comment and 1 area for change, one email that had 4 reinforcing comments and 8 areas for change). These emails were reviewed by 3 experienced interventionists (email text is presented in Multimedia Appendix 1).

### Measures

Respondents first reported their demographics (ie, age, gender, race, and ethnicity) and socioeconomic characteristics (ie, annual household income, highest level of education, and current employment status) using questions from the Behavioral Risk Factor Surveillance System [18].

Likelihood to self-monitor in the future was measured using 1 item (“How likely is it that you would continue to record your eating and physical activity in the online diary in the next week?”) with a 5-point Likert response scale (1=*very unlikely* to 5=*very likely*).

Motivation to make behavioral changes in the future was measured using 1 item (“How motivated would you be to continue to make behavioral changes in your eating and physical activity in the next week?”) with a 5-point Likert response scale (1=*not motivated* to 5=*very motivated*).

The perception of the counselor included 3 components, each measured by 1 item. The first 2 items were adapted from the Working Alliance Inventory Bond subscale [19]: “The counselor is genuinely concerned for my welfare” and “The counselor genuinely likes me.” Participants also responded to the following statement: “The counselor is understanding about my challenges.” Participants’ responses were recorded on a 5-point Likert scale (1=*strongly disagree* to 5=*strongly agree*).

The survey also captured impressions about 3 aspects of the emails, with items that inquired about the insightfulness of the feedback (“The e-mail gives me a new way of looking at weight and eating and physical activity behaviors,” adapted from the Working Alliance Inventory [19]), how tailored they perceived the email to be (“The information in the e-mail seems tailored,” adapted from a study by Valle et al [20]), and perceptions about the length of email (“I feel that the e-mail is... ‘too short’ to ‘too long’”). All items used a 5-point Likert response format.

### Independent Variables

The independent variables indicated the level of reinforcing comments (ie, 1 comment, 4 comments, and 8 comments) and the level of areas for change (ie, 1 area, 4 areas, and 8 areas). Interaction terms between the levels of reinforcing comments and areas for change were constructed to assess whether respondents’ likelihood to self-monitor and make behavioral changes in the future and their perceptions of the counselor and the email would change, given different combinations of feedback.

### Analyses

Demographic and socioeconomic characteristics were tabulated to examine the sample attributes. As each individual responded to all 9 email variations, mixed effects ordinal regression was used to model the 5-level ordinal responses. A random intercept for each individual was used. The model included the main effects of reinforcing comments, areas of change, and all two-factor interactions on respondents’ likelihood to self-monitor, motivation to make behavioral changes, and perceptions of the counselor and emails. The reference category for reinforcing comments is 1 reinforcing comment, and the reference category for areas for change is 1 area for change.

To effectively assess individuals’ perception of the self-monitoring feedback emails as well as for ease of interpretability, we calculated average predicted probabilities of the most positive category (ie, very likely, strongly agree, very motivated, and just right), conditional on the value of the random effect when the interaction between the 2 independent variables was included. Given that sociodemographic characteristics have been shown to affect self-monitoring rates [21-24], we adjusted the model to take into account age, ethnicity, race, gender, income, education level, employment status, and BMI. Analyses were conducted using STATA (version 16).

## Results

### Sample Characteristics

The demographic and socioeconomic characteristics of the participants are reported in Table 1. Approximately equal numbers of women and men participated, and most participants had at least some college education (74.0%). The sample was approximately evenly distributed between individuals with overweight and obesity.

**Table 1:** Sample sociodemographic characteristics (N=250).

Sociodemographic characteristics			Values, n (%)
**Age (years)**		
	18-24		10 (4.0)
	25-34		31 (12.4)
	35-44		46 (18.4)
	45-54		31 (12.4)
	55-64		33 (13.2)
	≥65		97 (38.8)
	Missing		2 (0.8)
**Gender**		
	Women		130 (52.0)
	Men		120 (48.0)
**Race**		
	White		127 (50.8)
	Black or African American		93 (37.2)
	Other		30 (12.0)
**Ethnicity**		
	Hispanic or Latino		23 (9.2)
**Educational level**		
	Lower than high school degree		64 (25.6)
	Some college		80 (32.0)
	Higher than college degree		105 (42.0)
	Missing		1 (0.4)
**Employed**			115 (46.0)
	**Annual household income (US $)**	
		≤24,999	54 (21.6)
		25,000-49, 999	73 (29.2)
		50,000-74,999	47 (18.8)
		≥75,000	75 (30.0)
		Missing	1 (0.4)
**BMI category (kg/m^2^)**		
	Obese (BMI≥30)		137 (54.8)
	Overweight (BMI=25.0-29.9)		113 (45.2)

### Likelihood to Self-Monitor in the Future

The odds that an individual expressed the likelihood to self-monitor in the future was at least 3 times higher after reading emails with 4 (odds ratio [OR] 3.22, 95% CI 2.18-4.75) or 8 reinforcing comments (OR 3.75, 95% CI 2.54-5.54) than after reading emails with 1 reinforcing comment (Table 2). In addition, the odds that individuals expressed a likelihood to self-monitor in the future was at least 2 times higher after reading emails with 4 (OR 2.24, 95% CI 1.52-3.28) or 8 areas for change (OR 2.58, 95% CI 1.75-3.80) than after reading emails with 1 area for change. The predictive margins on the interaction terms demonstrated that individuals reported the highest probability of being very likely to self-monitor in the future after reading the email with 4 reinforcing comments and 4 areas for change (49%, 95% CI 0.43-0.54; Table 3). Similar findings were observed with regard to the likelihood to self-monitor in the future based on levels of reinforcing comment, areas for change, and the interactions in the models that adjusted for sociodemographic characteristics and BMI category (Table 4).

**Table 2:** Odds ratios (with 95% CI) of different combinations of reinforcing comments and areas for change on all outcome measures (N=250).

Feedback content		Motivation, OR^a,b^ (95% CI)				Perception of the counselor, OR (95% CI)						Perception of the email, OR (95% CI)					
		Self-monitor in the future		Motivated to make behavior changes		Counselor concerned about welfare		Counselor likes me		Counselor understands challenges		Email gives insight		Email is tailored		Email length	
**Level of reinforcing comments (reference=1 reinforcing comment)**																	
	4 reinforcing comments		3.22 (2.18-4.75)		3.27 (2.24-4.78)		4.26 (2.91-6.22)		2.55 (1.73-3.76)		3.80 (2.62-5.50)		4.69 (3.23-6.83)		2.72 (1.89-3.92)		10.46 (7.01-15.62)
	8 reinforcing comments		3.75 (2.54-5.54)		4.76 (3.24-7.00)		4.98 (3.39-7.31)		3.11 (2.11-4.60)		5.32 (3.64-7.77)		5.70 (3.90-8.35)		3.50 (2.41-5.08)		17.65 (11.70-26.63)
**Level of areas for change (reference=1 area for change)**																	
	4 areas for change		2.24 (1.52-3.28)		2.27 (1.55-3.31)		3.13 (2.15-4.56)		2.34 (1.59-3.46)		3.12 (2.16-4.52)		3.81 (2.63-5.54)		2.62 (1.81-3.79)		13.61 (9.08-20.39)
	8 areas for change		2.58 (1.75-3.80)		2.52 (1.73-3.68)		4.61 (3.15-6.74)		2.50 (1.70-3.69)		4.45 (3.06-6.47)		5.01 (3.44-7.29)		3.40 (2.35-4.92)		107.55 (69.41-166.63)
**Interactions**																	
	4 reinforcing comments and 4 areas for change		0.58 (0.34-1.00)		0.57 (0.33-0.96)		0.37 (0.22-0.63)		0.54 (0.32-0.93)		0.49 (0.29-0.83)		0.33 (0.20-0.56)		0.39 (0.23-0.64)		0.13 (0.08-0.23)
	4 reinforcing comments and 8 areas for change		0.38 (0.22-0.66)		0.45 (0.27-0.77)		0.29 (0.17-0.49)		0.55 (0.32-0.95)		0.30 (0.18-0.50)		0.27 (0.16-0.46)		0.34 (0.20-0.57)		0.11 (0.07-0.19)
	8 reinforcing comments and 4 areas for change		0.44 (0.26-0.76)		0.44 (0.26-0.75)		0.48 (0.28-0.81)		0.54 (0.31-0.92)		0.30 (0.18-0.51)		0.29 (0.17-0.48)		0.36 (0.22-0.61)		0.24 (0.14-0.40)
	8 reinforcing comments and 8 areas for change		0.25 (0.15-0.43)		0.24 (0.14-0.41)		0.28 (0.16-0.47)		0.33 (0.19-0.58)		0.16 (0.10-0.28)		0.21 (0.12-0.36)		0.30 (0.18-0.51)		0.14 (0.08-0.23)

^a^OR: odds ratio.

^b^All odds ratios are significant at *P*<.05.

**Table 3:** Average predicted probabilities of combinations of email feedback on all outcome measures (N=250).^a^

Feedback content	Motivation, APP^b^ (95% CI)		Perception of the counselor, APP (95% CI)				Perception of the email, APP (95% CI)		
	Very likely to self-monitor in the future	Very likely to be motivated to make behavior changes	Strongly agree that the counselor is concerned about welfare	Strongly agree the counselor likes me	Strongly agree the counselor understands challenges	Strongly agree the email gives insight		Strongly agree the email is tailored	Length of the email is just right
1 reinforcing comment and 1 area for change	0.32 (0.27-0.36)	0.27 (0.23-0.32)	0.21 (0.17-0.25)	0.20 (0.16-0.24)	0.21 (0.17-0.26)	0.19 (0.15-0.24)		0.25 (0.21-0.30)	0.43 (0.37-0.48)
1 reinforcing comment and 4 areas for change	0.41 (0.35-0.46)	0.36 (0.31-0.41)	0.32 (0.27-0.37)	0.27 (0.23-0.32)	0.34 (0.29-0.39)	0.33 (0.29-0.38)		0.35 (0.30-0.40)	*0.65*^c^ (0.62-0.68)
1 reinforcing comment and 8 areas for change	0.42 (0.37-0.48)	0.37 (0.32-0.42)	0.36 (0.31-0.41)	0.28 (0.23-0.32)	0.38 (0.33-0.43)	0.37 (0.32-0.42)		*0.39*^c^ (0.33-0.44)	0.45 (0.40-0.50)
4 reinforcing comments and 1 area for change	0.45 (0.39-0.51)	0.40 (0.35-0.45)	0.35 (0.31-0.40)	0.28 (0.24-0.32)	0.36 (0.31-0.41)	0.36 (0.31-0.41)		0.36 (0.31-0.41)	*0.65*^c^ (0.62-0.68)
4 reinforcing comments and 4 areas for change	*0.49*^c^ (0.43-0.54)	0.43 (0.37-0.49)	0.37 (0.32-0.42)	0.30 (0.26-0.35)	*0.42*^c^ (0.36-0.47)	0.39 (0.34-0.44)		0.36 (0.31-0.41)	0.64 (0.60-0.67)
4 reinforcing comments and 8 areas for change	0.45 (0.39-0.51)	0.42 (0.36-0.47)	0.39 (0.34-0.44)	0.31 (0.27-0.36)	0.34 (0.35-0.45)	*0.40*^c^ (0.35-0.45)		0.38 (0.32-0.43)	0.43 (0.38-0.48)
8 reinforcing comments and 1 area for change	0.47 (0.41-0.53)	*0.45*^c^ (0.39-0.50)	0.37 (0.32-0.43)	0.30 (0.25-0.34)	0.41 (0.35-0.46)	0.38 (0.33-0.44)		*0.39*^c^ (0.34-0.44)	0.64 (0.61-0.67)
8 reinforcing comments and 4 areas for change	0.47 (0.41-0.53)	*0.45*^c^ (0.39-0.50)	*0.42*^c^ (0.37-0.48)	*0.32*^c^ (0.27-0.37)	0.40 (0.34-0.45)	0.39 (0.34-0.45)		0.38 (0.33-0.44)	0.54 (0.50-0.58)
8 reinforcing comments and 8 areas for change	0.42 (0.36-0.47)	0.38 (0.33-0.44)	0.40 (0.35-0.46)	0.28 (0.24-0.33)	0.36 (0.31-0.41)	0.39 (0.34-0.44)		*0.39*^c^ (0.34-0.45)	0.32 (0.28-0.37)

^a^All probabilities were significant at the *P*<.01 level.

^b^APP: average predicted probabilities.

^c^Highest probability (or probabilities) is italicized.

**Table 4:** Adjusted odds ratios for sociodemographic factors on all outcome measures (N=250).^a^

Characteristics			Motivation, OR^b^ (95% CI)		Perception of the counselor, OR (95% CI)				Perception of the email, OR (95% CI)		
			Self-monitor in the future	Motivated to make behavior changes	Counselor concerned about welfare	Counselor likes me	Counselor understands challenges	Email gives insight		Email is tailored	Email length
**Level of reinforcing comments (reference=1)**											
	4 reinforcing comments		3.22^***c^ (2.18-4.77)	3.34^***^ (2.28-4.91)	4.33^***^ (2.96-6.35)	2.50^***^ (1.69-3.70)	3.76^***^ (2.59-5.47)	4.68^***^ (3.21-6.82)		2.78^***^ (1.92-4.02)	10.64^***^ (7.10-15.94)
	8 reinforcing comments		3.87^***^ (2.61-5.73)	4.74^***^ (3.21-6.99)	5.03^***^ (3.42-7.41)	3.22^***^ (2.17-4.78)	5.57^***^ (3.79-8.18)	5.75^***^ (3.92-8.45)		3.68^***^ (2.52-5.36)	18.26^***^ (12.04-27.68)
**Level of areas for change (reference=1)**											
	4 areas for change		2.25^***^ (1.53-3.32)	2.28^***^ (1.56-3.34)	3.08^***^ (2.12-4.50)	2.27^***^ (1.54-3.36)	3.16^***^ (2.18-4.58)	3.82^***^ (2.62-5.56)		2.62^***^ (1.80-3.80)	13.95^***^ (9.27-21.01)
	8 areas for change		2.52^***^ (1.71-3.72)	2.53^***^ (1.73-3.70)	4.58^***^ (3.13-6.72)	2.54^***^ (1.72-3.76)	4.59^***^ (3.14-6.69)	5.04^***^ (3.45-7.36)		3.52^***^ (2.42-5.10)	114.68^***^ (73.61-178.65)
**Interactions**											
	4 reinforcing comments and 4 areas for change		0.59^*^ (0.34-1.01)	0.56^**^ (0.33-0.95)	0.38^***^ (0.22-0.64)	0.58^*^ (0.34-1.01)	0.51^**^ (0.30-0.85)	0.34^***^ (0.20-0.56)		0.39^***^ (0.24-0.66)	0.14^***^ (0.08-0.24)
	4 reinforcing comments and 8 areas for change		0.40^***^ (0.23-0.69)	0.45^***^ (0.26-0.77)	0.29^***^ (0.17-0.49)	0.57^**^ (0.33-0.98)	0.30^***^ (0.18-0.50)	0.28^***^ (0.17-0.47)		0.33^***^ (0.20-0.56)	0.11^***^ (0.06-0.19)
	8 reinforcing comments and 4 areas for change		0.43^***^ (0.25-0.75)	0.44^***^ (0.26-0.76)	0.48^***^ (0.28-0.82)	0.56^**^ (0.32-0.96)	0.30^***^ (0.18-0.51)	0.29^***^ (0.17-0.49)		0.35^***^ (0.21-0.59)	0.24^***^ (0.14-0.42)
	8 reinforcing comments and 8 areas for change		0.24^***^ (0.14-0.42)	0.23^***^ (0.14-0.40)	0.27^***^ (0.16-0.46)	0.31^***^ (0.18-0.54)	0.15^***^ (0.09-0.26)	0.21^***^ (0.12-0.36)		0.28^***^ (0.16-0.47)	0.13^***^ (0.08-0.23)
**Sociodemographic characteristics**											
	**Age (reference: 18-24 years), years**										
		25-34	11.96^**^ (1.46-98.04)	13.21^**^ (1.82-95.95)	18.40^***^ (2.71-124.84)	16.51^***^ (1.99-136.78)	7.25^**^ (1.26-41.73)	10.30^**^ (1.72-61.56)		9.56^**^ (1.43-63.72)	0.77 (0.22-2.75)
		35-44	7.65^*^ (1.00-58.65)	11.83^**^ (1.73-80.80)	17.33^***^ (2.71-110.77)	10.70^**^ (1.38-82.97)	6.46^**^ (1.19-35.18)	6.25^**^ (1.11-35.27)		3.17 (0.51-19.82)	1.36 (0.40-4.67)
		45-54	3.16 (0.39-25.66)	6.80^*^ (0.94-49.01)	17.72^***^ (2.63-119.48)	6.53^*^ (0.80-53.58)	5.25^*^ (0.92-30.07)	5.62^*^ (0.95-33.32)		5.03^*^ (0.76-33.22)	0.74 (0.21-2.63)
		55-64	4.33 (0.53-35.71)	8.42^**^ (1.15-61.68)	29.52^***^ (4.30-202.73)	7.66^*^ (0.92-63.79)	9.05^**^ (1.56-52.46)	13.14^***^ (2.17-79.42)		5.79^*^ (0.87-38.75)	1.04 (0.29-3.73)
		≥65	4.62 (0.62-34.41)	9.10^**^ (1.37-60.50)	18.46^***^ (2.96-115.02)	6.82^*^ (0.90-51.46)	6.46^**^ (1.22-34.33)	9.17^**^ (1.66-50.53)		2.66 (0.44-16.18)	0.87 (0.26-2.94)
	**Hispanic or Latino (reference: Non-Hispanic)**		1.54 (0.42-5.72)	1.45 (0.42-5.01)	1.349 (0.41-4.45)	1.40 (0.38-5.16)	0.88 (0.30-2.62)	1.15 (0.38-3.53)		1.01 (0.31-3.30)	0.50^*^ (0.23-1.11)
	**Race (reference: White)**										
		Black or African American	4.34^***^ (1.79-10.51)	4.05^***^ (1.75-9.39)	2.32^**^ (1.03-5.23)	1.79 (0.74-4.32)	2.06^*^ (0.98-4.32)	3.00^***^ (1.40-6.43)		1.02 (0.46-2.27)	0.89 (0.52-1.53)
		Other	0.74 (0.21-2.62)	1.166 (0.35-3.85)	0.99 (0.31-3.13)	0.79 (0.22-2.77)	1.07 (0.38-3.07)	1.40 (0.47-4.11)		0.89 (0.28-2.79)	1.13 (0.52-2.42)
	**Gender (reference: men)**										
		Women	1.49 (0.70-3.16)	2.00^*^ (0.98-4.08)	2.32^**^ (1.17-4.62)	2.09^*^ (0.98-4.42)	2.38^***^ (1.27-4.48)	2.39^***^ (1.25-4.55)		1.86^*^ (0.94-3.69)	0.89 (0.56-1.40)
	**Annual household income (reference: ≤24,999), US $**										
		25,000-49,999	1.19 (0.43-3.27)	1.15 (0.44-3.01)	0.86 (0.34-2.17)	1.05 (0.38-2.92)	0.86 (0.37-2.00)	0.89 (0.37-2.12)		0.56 (0.22-1.41)	1.35 (0.73-2.49)
		50,000-74,999	2.74^*^ (0.84-9.01)	1.61 (0.52-4.97)	1.34 (0.45-3.95)	1.49 (0.45-4.89)	1.09 (0.41-2.93)	1.70 (0.62-4.68)		1.03 (0.35-3.03)	1.318 (0.64-2.71)
		≥75,000	3.73^**^ (1.22-11.43)	2.32 (0.80-6.72)	2.70^*^ (0.97-7.52)	2.34 (0.76-7.17)	2.53^*^ (0.99-6.44)	2.52^*^ (0.97-6.56)		1.93 (0.70-5.34)	0.93 (0.47-1.83)
	**Educational level (reference: high school degree or less)**										
		Some college	0.46 (0.18-1.17)	0.64 (0.26-1.54)	0.76 (0.33-1.79)	0.56 (0.22-1.41)	0.55 (0.25-1.19)	0.59 (0.27-1.32)		1.10 (0.47-2.56)	0.79 (0.45-1.39)
		College degree or more	0.78 (0.30-2.04)	0.89 (0.36-2.23)	1.04 (0.43-2.51)	0.54 (0.21-1.42)	0.74 (0.33-1.65)	0.67 (0.29-1.54)		1.15 (0.48-2.76)	0.58^*^ (0.32-1.04)
	**Employment status (reference: not employed)**										
		Employed	0.61 (0.24-1.53)	0.67 (0.28-1.61)	1.26 (0.54-2.95)	2.22^*^ (0.88-5.61)	1.46 (0.67-3.16)	1.40 (0.64-3.10)		1.27 (0.55-2.93)	1.25 (0.71-2.18)
	**BMI category (reference: overweight)**										
		Obese	0.50^*^ (0.24-1.04)	0.42^**^ (0.21-0.85)	0.60 (0.31-1.18)	0.81 (0.39-1.69)	0.62 (0.33-1.14)	0.72 (0.38-1.34)		0.63 (0.33-1.23)	0.83 (0.53-1.27)

^a^Adjusted for sociodemographic characteristics (ie, age, ethnicity, race, gender, income, education level, employment status, and BMI category).

^b^OR: odds ratio.

^c^*P<.1, **P<.05, ***P<.01.

Overall, individuals with certain sociodemographic characteristics had significantly higher odds of reporting a likelihood to self-monitor in the future (Table 4); these characteristics were identifying as Black or African American (OR 4.34, 95% CI 1.79-10.51), having an annual household income of US $50,000 to US $74,999 (OR 2.74, 95% CI 0.84-9.01) or US $75,000 or more (OR 3.73, 95% CI 1.22-11.43), and aged 25 to 44 years (OR 7.65-11.96). In addition, those with obesity (OR 0.50, 95% CI 0.24-1.04) were less likely to report a likelihood to self-monitor in the future, compared with those with overweight.

### Motivation to Make Behavioral Changes

The odds that individuals would be motivated to make behavioral changes was 3 to almost 5 times higher after reading emails with 4 (OR 3.27, 95% CI 2.24-4.78) or 8 (OR 4.76, 95% CI 3.24-7.00) reinforcing comments than after reading emails with 1 reinforcing comment, and the odds that individuals would be motivated to make behavioral changes was at least 2 times higher after reading emails with 4 (OR 2.27, 95% CI 1.55-3.31) or 8 (OR 2.52, 95% CI 1.73-3.68) areas for change (Table 2). The predictive margins on the interaction terms demonstrated that individuals reported the highest probability of being very likely to be motivated to make behavioral changes after reading the email with 8 reinforcing comments and 1 area for change (45%; 95% CI 0.39-0.50; Table 3) or the email with 8 reinforcing comments and 4 areas for change (45%; 95% CI 0.39-0.50).

Again, there were similar outcomes in the models that adjusted for sociodemographic characteristics and BMI category (Table 4). Those who identified as Black or African American (OR 4.05, 95% CI 1.75-9.39) or as a woman (OR 2.00, 95% CI 0.98-4.08) were more likely to report being motivated to make behavioral changes (Table 4). Respondents older than 24 years had significantly higher odds to report being motivated to make behavioral changes (ORs 6.80-13.21) than those aged 18-24 years. Respondents with obesity (OR 0.42, 95% CI 0.21-0.85) were less likely to report being motivated to make behavioral changes compared with those who were overweight.

### Perception of Counselor

The odds that individuals rated the counselor as concerned for their welfare was 4-5 times higher after reading emails with 4 (OR 4.26, 95% CI 2.91-6.22) or 8 (OR 4.98, 95% CI 3.39-7.31) reinforcing comments than after reading those with 1 reinforcing comment, and 3-4 times higher after reading emails with 4 (OR 3.13, 95% CI 2.15-4.56) or 8 (OR 4.61, 95% CI 3.15-6.74) areas for change (Table 2) than after reading emails with 1 area for change. In addition, the odds that individuals indicated that the counselor genuinely liked them were significantly higher after reading emails with 4 (OR 2.55, 95% CI 1.73-3.76) or 8 (OR 3.11, 95% CI 2.11-4.60) reinforcing comments or 4 (OR 2.34, 95% CI 1.59-3.46) or 8 (OR 2.50, 95% CI 1.70-3.69) areas for change than after reading emails with only one reinforcing comment or 1 area for change. Furthermore, the odds that individuals reported that the counselor understands their challenges was significantly higher after reading emails with 4 (OR 3.80, 95% CI 2.62-5.50) or 8 (OR 5.32, 95% CI 3.64-7.77) reinforcing comments or 4 (OR 3.12, 95% CI 2.16-4.52) or 8 (OR 4.45, 95% CI 3.06-6.47) areas for change.

The interactions indicated that individuals had the highest average probability of strongly agreeing that the counselor was genuinely concerned for their welfare (42%; 95% CI 0.37-0.48) after reading emails with 8 reinforcing comments and 4 areas for change (Table 3). In addition, individuals had the highest average probability of strongly agreeing with the counselor genuinely liked them (32%; 95% CI 0.27-0.37) after reading emails with 8 reinforcing comments and 4 areas for change. As for the perception of the counselor being understanding of their challenges, the interactions demonstrated that individuals had the highest average probability of strongly agreeing after reading emails with 4 reinforcing comments and 4 areas for change (42%; 95% CI 0.36-0.47). The sociodemographic group differences in perceptions of the counselor were similar to those described earlier (Table 4).

### Perception of the Emails

Perceptions of insightfulness, tailoring, and length of the emails were significantly more favorable after reading emails with 4 or 8 reinforcing comments or 4 or 8 areas for change than after reading emails with only one reinforcing comment or 1 area for change (Table 2). The interactions demonstrated that individuals had the highest average probability of strongly agreeing that the emails gave them a new way of looking at diet and physical activity behaviors (40%; 95% CI 0.35-0.45) after reading emails with 4 reinforcing comments and 8 areas for change (Table 3). Perceptions that the emails were tailored were strongest (39%; 95% CI 0.33-0.44) after reading emails with 1 reinforcing comment and 8 areas for change, 8 reinforcing comments and 1 area for change, and 8 reinforcing comments and 8 areas for change. Finally, perceptions that the length of the emails being just right were the highest (65%; 95% CI 0.62-0.68) after reading emails with 4 reinforcing comments and 1 area for change and emails with 1 reinforcing comment and 4 areas for change. The sociodemographic differences in the perception of emails are similar to those described earlier (Table 4).

## Discussion

This study conducted a factorial experiment to examine the perception of email feedback on diet and physical activity behaviors among individuals with overweight or obesity and a desire to lose weight. The main effects showed that the emails with more reinforcing comments or areas for change were better received, in general, with often a small incremental benefit for 8 reinforcing comments or areas for change versus 4 reinforcing comments or areas for change. Thus, participants may view longer messages that provide the overall context of their behaviors over a week as more positive, rather than shorter messages containing just one reinforcing comment or area for change. The interactions indicated that the best combination for 3 of 8 dimensions assessed was the email with 8 reinforcing comments and 4 areas for change, which was associated with higher ratings of motivation to make behavioral changes in the future, counselor’s concern for their welfare, and perceptions that the counselor genuinely likes them. Nonetheless, as consistent self-monitoring is important for effective weight management [12], perhaps the best combination among those tested in this study is the email with 4 reinforcing comments and 4 areas for change because it resulted in the highest average probability of reporting being very likely to self-monitor in the future if continuing to self-monitor is weighted most heavily.

Interestingly, the longest email combinations (ie, the 8 reinforcing comments and 8 areas for change, the 1 reinforcing comment and 8 areas for change, and the 8 reinforcing comments and 1 area for change combinations) had the highest average probability of being rated as being tailored. Perhaps, individuals viewed more feedback as being more customized to their situation. In contrast, the email combination that had the maximum number of areas of change (ie, 8) and a moderate number of reinforcing comments (ie, 4) had the highest likelihood of being viewed as insightful, indicating that insightfulness might be perceived as a balance between reinforcing comments and areas for change. However, the emails judged as *just right* in length were shorter than any of these options (ie, the 1 reinforcing comment and 4 areas for change and the 4 reinforcing comments and 1 area for change combinations). Thus, the optimal constellation of areas of change and number of reinforcing comments may depend on the outcome, and there are few data to guide which of these outcomes is associated with consistent self-monitoring and, ultimately, superior weight loss.

The responses to the different email feedback combinations varied very little across the demographic groups considered, which is interesting as some groups have been found to have significantly higher or lower self-monitoring engagement in previous studies. For example, studies have found that women have lower engagement with self-monitoring than men [21,22], whereas we found that women and men in this study reported a similar likelihood to self-monitor in the future, although it must be acknowledged that these were hypothetical projections rather than actual self-monitoring behavior. In addition, previous research indicates that older individuals are more likely to engage in self-monitoring than younger individuals [21,23,24], although we found that individuals aged 25 to 44 years had a higher odds to report that they were likely to self-monitor in the future than individuals who were aged 18 to 24 years and the older age groups did not differ from the youngest group. This is in contrast to previous research, which found that older adults (ie, aged 60-85 years) were more successful in losing weight in the Diabetes Prevention Program’s weight management intervention [25], perhaps because older individuals have fewer competing obligations. Finally, although Black respondents indicated that they would be more likely to self-monitor in the future, previous research has not found significant race-based differences in self-monitoring engagement [17,21]. Nonetheless, a meta-analysis that examined the impact of tailored messages for health behavioral change in general also found a minimal impact of sociodemographic factors on the effect of the messages [5]. It will be important to examine whether responses to the different email feedback combinations are comparable across demographic groups when studying actual self-monitoring behavior, rather than self-reported likelihood of self-monitoring in the future.

There are some limitations to this study, which merit acknowledgment. Importantly, participants who rated the emails were not enrolled in a weight loss study and thus provided hypothetical responses to feedback emails that were not personalized to the individual in this study. It is unknown if the likelihood of self-monitoring or motivation to make behavioral changes in the future would translate to actual behavior. However, all respondents were either overweight or obese and expressed an interest in losing weight, so it may be assumed that the sample resembled respondents who would join a weight loss program to some extent. In addition, the concepts were each measured by a single item. Although these single items were adapted from previous studies when possible and have face validity, the psychometric properties of the items were not examined. Future studies should examine individuals actively engaged in weight loss efforts to determine whether the composition of emailed feedback drives actual self-monitoring patterns and ultimately influences weight loss outcomes. Furthermore, we arbitrarily chose the 3 levels of each variable (ie, 1, 4, and 8) so that there was separation between each level and to represent what might be considered a small number of comments, a moderate number of comments, and a large number of comments. Furthermore, we tested only 3 levels of each variable, and thus, it is possible that the *ideal* combination was not examined in this study. In future research, it will be important to test other levels of the variables, including 0 reinforcing comments or areas for change; however, researchers need to be aware of participant burden and attention limitations, which is why we limited our survey to 9 emails. Despite these limitations, to our knowledge, this is the first study to explore perspectives on self-monitoring feedback and to provide the first indication of the *sweet spots* in providing weekly self-monitoring feedback. An additional strength is the diversity of the sample with respect to gender and race and ethnicity, which allowed some insights along those dimensions.

Given the broad and escalating need for obesity treatment, it is essential to optimize treatment components for efficacy and cost-effectiveness, including self-monitoring feedback. A recent study reported that the average time for counselors to review self-monitoring records and compose weekly dietary and physical activity self-monitoring feedback emails is approximately 28 min, following a guideline of 6 reinforcing comments and 3 areas for change [26]. Some have opted to use computer-driven algorithms to generate feedback [27], whereas others report that human-generated feedback produces superior weight loss outcomes [4]. It will be important in future research to examine whether the optimal approach for self-monitoring feedback differs in the context of human-generated emails compared with algorithm-driven emails and to identify the most time-efficient (and therefore least costly) feedback constellation for humans to generate. This study offers some specific hypotheses to test and, as such, can advance this critical aspect of behavioral weight management intervention refinement.

## Appendix

Multimedia Appendix 1 The 9 emails used in this research. Blue text indicates the combination of the number of reinforcing comments and areas for change. Red text indicates an area for change, and green text indicates a reinforcing comment. Black text is standard for every email.

## Abbreviations

OR: odds ratio
