# Supplementary material for: Importance of Multiple Reinforcing Comments and Areas for Change in Optimizing Dietary and Exercise Self-Monitoring Feedback in Behavioral Weight Loss Programs: Factorial Design
Source: J Med Internet Res. 2020 Nov 23;22(11):e18104. doi: 10.2196/18104 (PMC7685695; doi:10.2196/18104)
Supplement: Multimedia Appendix 1 [file jmir_v22i11e18104_app1.docx]

# The 9 emails used in this research, with blue text indicating the combination of the number of reinforcing comments and areas for change. Red text indicates an area for change, and green text indicates a reinforcing comment. Black text is standard for every email.

# Reinforcing Comments 1, Areas for Change 1

Dear Participant,

Great job self-monitoring this week!

- It looks like you aren’t much of a breakfast fan—how, if at all, is that working for you? Or are you finding that you are very hungry at lunch time?
- Great portion control with the almonds—it can be hard to stop at 10!

Please let me know if you have any questions! Have a great week!

Your Counselor

# Reinforcing Comments 1, Areas for Change 4

Dear Participant,

Great job self-monitoring this week!

- Looking back on your KFC meal which put you over your calorie limit on Tuesday, what might have been a change that you might have made that could have helped you meet your calorie goal that day (e.g., no biscuit)?
- The 3 slices of pizza on the 28th probably sent you over your calorie goal—what pairing (e.g., salad, steamed veggies) with the pizza might have been possible to help you reduce your portion of pizza?
- Your snacks on Monday and Wednesday added quite a few calories—what, if anything, might have helped to reduce the number of snacks you ate on these days or made them healthier choices?
- What strategies do you think might have helped in that situations to reduce your calories/fat from dessert on Friday?
- You did well with managing portion sizes of higher calorie items throughout the week (e.g., cookie at KFC, buttered mashed potatoes, cheese, meatballs, chocolates).

Please let me know if you have any questions! Have a great week!

Your Counselor

# Reinforcing Comments 1, Areas for Change 8

Dear Participant,

Great job self-monitoring this week!

- On your challenging food days this past week, what did you learn that might help you make different choices in the future when similar situations come up (e.g., pair pizza with a salad and have a smaller portion of the pizza, switch snacks to those with more fiber and protein to give you greater fullness, ask for two portions of carrots rather than potatoes at Wolfgang’s)?
- What modifications do you think might help you come closer to your fat goal (e.g., switch to lower-fat cheese, consider lower-fat hotdogs, put less dressing or a lower-fat dressing on salads)?
- What might you pair with yogurt on busy work days, so that you aren’t so very hungry by dinner time (e.g., portion-controlled meals)?
- It looks like calories from alcohol and sweets may have sent you over your goal on a few of the days. Are there strategies that you might consider to cut out some of these calories (e.g., making spritzers with wine, beer, or margaritas by adding seltzer water or diet soda and half of the alcohol, adding a fruit salad to dessert and reduce the portion size of the ice cream/cheesecake)?
- Were you surprised by how much fat there was in the egg salad sandwich? Typically sandwiches that aren't mayo-based (and don't have much if any cheese) are lower in calories. Do you think there are alternatives that might be lower fat that might work for you?
- What strategies do you think would have been helpful to manage the Outback meal to trim some calories (e.g., having a side of vegetables rather than fries, asking to not receive the bread and butter, having less dressing on the salad)?
- It seems like the goldfish crackers might be a challenge to eat in small portion sizes -- are there alternatives (e.g., popcorn, carrots with hummus) that you think might also work for you?
- Chips at Tex-Mex places can be a big challenge for lots of folks-- how would you like to manage the chips in the future?
- Great job fitting in exercise when you can—and taking the future weather into account, so you can make sure to fit it in.

Please let me know if you have any questions! Have a great week!

Your Counselor

# Reinforcing Comments 4, Areas for Change 1

Dear Participant,

Great job self-monitoring this week!

- There were a few days where you went over your fat goal (Monday, Tuesday, and Thursday)—is the fat goal one that you are interested in trying to meet? If so, some foods that you ate which would have impacted your total fat intake the most, in this past week, were: the bratwurst, the chicken leg (dark meat tends to be higher in fat than white meat in a breast), the crustless quiches, and the chocolate chip cookie.
- You met your calorie goal on all but one day, and seem to maintain an even level of calories across the days – nice work!
- Great job getting a good start to the day with a high protein/high fiber breakfast on most days!
- Great job choosing the apple at Panera with your salad instead some of the other higher calorie and lower fiber side options!
- Nice work fitting in exercise and strength training!

Please let me know if you have any questions! Have a great week!

Your Counselor

# Reinforcing Comments 4, Areas for Change 4

Dear Participant,

Great job self-monitoring this week!

- Were you surprised by the calories/fat in the oatmeal cookies on Friday and Saturday? How might you want to handle this food situation in the future?
- What ideas do you have for substitutions for potato salad or burgers, or would you or the cook in your house be interested in some substitution ideas for potato salad and burgers?
- What do you think about some of the chip alternatives that can reduce fat intake-- for example, popcorn or baked chips?
- I know you mentioned that last weekend was a food challenge because you were entertaining-- when you look back on the situations, what strategies, if any, do you see that you could try in a similar situation in the future?
- Great job fitting in a nice variety of fruits and vegetables again this week!
- Great choices of lean protein (e.g., egg, grilled chicken, yogurt, turkey).
- Great choice of a wine spritzer on Sunday-- that's a great way to limit adult beverage calories! :-)
- Great exercise throughout the week!

Please let me know if you have any questions! Have a great week!

Your Counselor

# Reinforcing Comments 4, Areas for Change 8

Dear Participant,

Great job self-monitoring this week!

- Were you surprised by the calories in the pizza, the potatoes, the broccoli cheddar soup, and coconut shrimp? What ideas do you have for cutting the portion of these higher calorie foods or choosing alternatives?
- Fruits and vegetables can be a good way of filling up with few calories like you did for lunch on Tuesday—what other times, if any, could you add fruits and vegetables that might be helpful?
- It seems like sweets could be challenging for you—did I get this right, or is this an unusual week for you?
- It looks like you might have had some challenging restaurant dinners on Saturday and Sunday—what, if any, strategies, are you thinking might help in the future?
- Sometimes it can help to pull out all of the portion size tools—cups, spoons, and food scale (if you have one) and see if you can pick out any portions that you might be underestimating; what would you think about trying this experiment?
- On the days, where there is no dinner listed, did you skip dinner, or were you not able to self-monitor?
- You went over your calorie goal on Monday—on this same day you had several Clif Bars; are you finding these satisfying or might this be one reason you went over your goal?
- Although you met your calorie goal, your fat goal seems like it is still a challenge; what do you think might be some feasible ways of trimming fat (e.g., reducing the amount of dressing on salads, getting lower fat hot dogs, mayonnaise or cheese, swapping the fries for a fruit cup at Chik-fil-A, choosing a different Subway sandwich, or ordering choosing grilled shrimp or chicken dishes at Chinese restaurants).
- It looks like you are finding greek yogurt, protein bars, and cheese sticks to be sources of lean protein that work for you.
- Great choice of whole grain items (e.g., oatmeal, whole wheat tortillas, whole wheat bread)-- it looks like you make that choice at least once per day!
- Good choices of broth-based soup—that can be a great way of filling up for few calories.
- Great job with your weight loss already—9lbs; we know that those who lose weight early on in the program tend to do better in the long term!

Please let me know if you have any questions! Have a great week!

Your Counselor

# Reinforcing Comments 8, Areas for Change 1

Dear Participant,

Great job self-monitoring this week!

- How are you thinking that you will want to manage the situation with the sugar cookies in the future? If you want to continue to make them for your grandkids, how do you think that you can handle this situation without feeling like you overindulged?
- Great job meeting your calorie goal on most days during the week!
- You did a great job fitting in lean protein (i.e., chicken, milk, yogurt, high protein oatmeal, turkey, low-fat cheese, tofu, black bean and lentil soup)!
- Great job choosing low or no calorie beverages most of the time!
- Good portion control on the pizza and ice cream on Wednesday!
- Nice pairing of your steak with a salad on Friday.
- The raisin bran cereal that you chose several times this week can be a great way to fit in fiber, that helps you feel full!
- Great portion control with the lobster sandwich on Sunday-- having half for lunch and half for dinner.
- Nice cardio and strength training throughout the week!

Please let me know if you have any questions! Have a great week!

Your Counselor

# Reinforcing Comments 8, Areas for Change 4

Dear Participant,

Great job self-monitoring this week!

- What alternatives do you think might have worked for the potato lasagna? Like you noted, this was more than half of your fat for the day!
- The 3 slices of pizza on Saturday probably put you over your fat goal—what, if any, might you pair with the pizza that might help you choose 1-2 slices?
- It looks like dinners on the weekdays and lunches on the weekend days were maybe a challenge this week—how typical was that? If typical, what alternatives do you think might you consider?
- What substitutions are you thinking about to help meet your fat goals (e.g., turkey hotdogs, salad with lower calorie dressing rather than coleslaw, avoiding fries).
- Good job fitting in lots of low or no-calorie beverages!
- Great choice with the oatmeal on Monday-- the protein, fiber, and water in it makes it filling!
- Great job grabbing a banana for a snack on Tuesday!
- Nice pairing of meals with fruit (i.e., oatmeal with grapefruit on Wednesday and Thursday and an apple with your sandwich on Friday).
- Nice pairing of your yogurt with your sandwich—how, if at all, does this additional protein help you stay satisfied until dinner?
- Good choice of the grilled nuggets over the fried variety on Wednesday and pairing this meal with a fruit cup (and keeping the fries to a small!).
- Good job avoiding the cheese on the burger at the ballpark!
- Great job fitting in lots of walks this week!

Please let me know if you have any questions! Have a great week!

Your Counselor

# Reinforcing Comments 8, Areas for change 8

Dear Participant,

Great job self-monitoring this week!

- I am surprised with the calories and fat in the Greek Salad on Tuesday—if you think that the calories/fat are accurate, what would you think might work for you to reduce calories and fat (e.g., dressing on the side, or reducing the portion of dressing)?
- The fat and protein in the chicken soup on Wednesday is surprising-- typically broth-based soups are much lower in fat, and they are typically higher in protein; what alternative soup, if any, might you try, or do you think it was an error in the entry?
- Sushi can often be a healthier choice-- what modifications do you think you could make, that you would still enjoy, in order to reduce the calories and fat (e.g., shrimp without the crunchy topping, a roll that doesn't have the amount of sauce a firecracker roll typically does)?
- It looks like your meal at Panera may have been a challenge in meeting your calorie goals. What other options at Panera might work for you (for example, the choose-two option at Panera, where you might try a salad together with half of a sandwich)?
- Would there be a way, do you think, of making Zaxby's possible to fit within your calorie and fat goals (e.g., grilled chicken, pairing a small amount of chicken with a salad), or do you think it would be better to choose a different restaurant?
- What kind of meal plan, if any, do you think might work for you, to spread your calories throughout the day? Some people decide on a certain number of calories per meal (for example, 400 kcal for breakfast, 400 kcal for lunch, 400 kcal for dinner, and then 150 kcal for a morning snack and 150 kcal for an afternoon snack).
- It looks like the chocolate chip cookies sent you over your calorie and fat goals on Thursday—what do you think might be strategies to manage the portion with these in the future?
- How did it go with having just one slice of pizza at Mellow Mushroom? Were you satisfied by your portion? I wonder whether there are alternative types of pizza there that might be lower-fat and potentially more satisfying.
- Great job coming close, on average, to your calorie goal!
- Great job having a fruit or vegetable at most meals this week!
- Good choice of whole grain cereal on Tuesday-- with protein and fiber!
- Good job managing the portion of your chicken fingers by saving some for the next day!
- Good job choosing lean protein for your tacos on Thursday.
- Great job fitting in strength training and flexibility training (i.e., yoga) this week!
- The Dannon smoothie sounds like a great lean protein snack!
- Great choices at Cracker Barrel-- chicken with vegetables! Cracker Barrel can be challenging—but it looks like you found a healthy option!
- Great choice of the turkey burger and veggies on Saturday—you saved yourself quite a few calories by choosing a leaner meat and veggies instead of fries!

Please let me know if you have any questions! Have a great week!

Your Counselor
